# Reappearance of Menstruation after Parturition

**Published:** 1916-03

**Authors:** 


**Reappearance of Menstruation After Parturition.** Hugo Ehrenfest, St. Louis, quoted in Old Dominion Jour. of Med. and Surg., Dec., from observation of 309 births among 209 women, concludes that menstruation recurs within 12 weeks in 50% of cases and before lactation is completed in over 80% Among primiparae, the percentage is still greater.

